# Supplementary material for: Multimodal imaging and functional analysis of the chick NMDA retinal damage model
Source: PLoS One. 2021 Sep 7;16(9):e0257148. doi: 10.1371/journal.pone.0257148 (PMC8423281; doi:10.1371/journal.pone.0257148)
Supplement: S1 File — (ZIP) [file pone.0257148.s004.zip › SD-OCT/C200727/017/D21-Pwk4/C200727--017_OS_V_14X14_0_REGAVG0000075/Manual_PreDefineFiveByFiveGridRectangularRetina_1.pdf]

Five By Five Grid Template Report

**Patient ID**	C200727	**Name**	See InVivoVue
**Exam Date**	See InVivoVue	**Exam Time**	See InVivoVue
**Eye**	OS	**Averaged**	N/A
**Depth (mm)**	1.65	**Depth Samples**	1024
**Pitch (um/pix)**	35.000 x 35.000 x 1.608	**Refractive Index**	See InVivoVue
**Scan Type**	Rectangular 14.000mm x 14.000mm x 1.647mm , 400 x 400 x 1024		

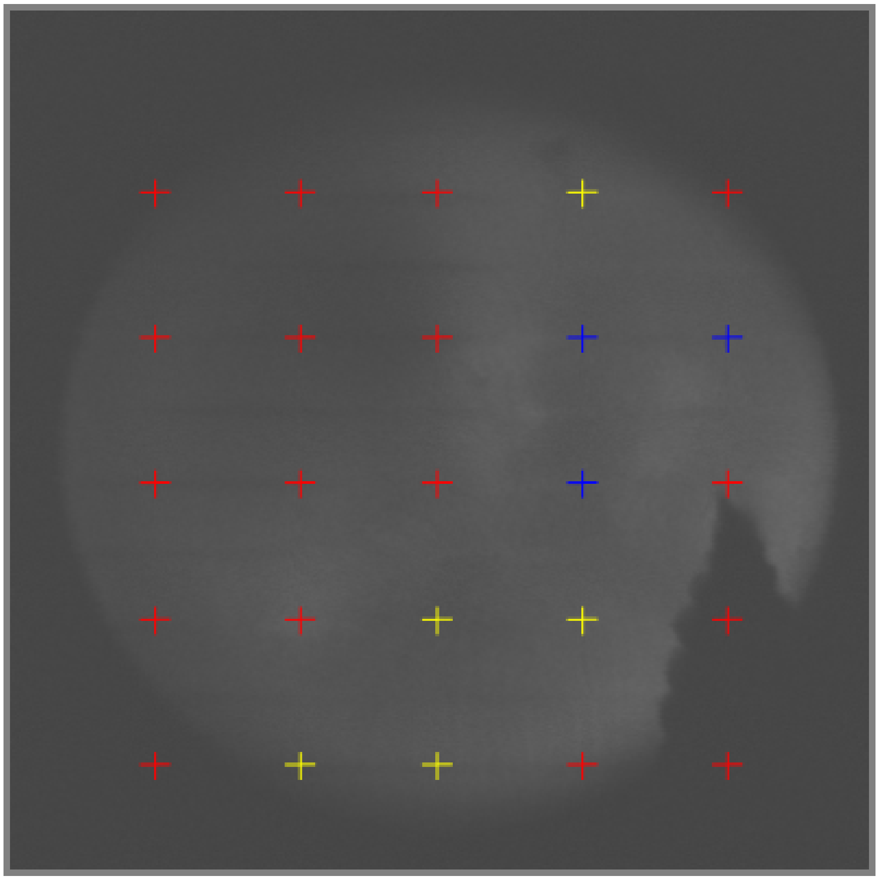

Thickness Metrics

Analysis	Average(mm)	Minimum(mm)	Maximum(mm)	STDEV(mm)
**IRNFL - RPE**	0.2756	0.2702	0.2808	0.0053
**IRNFL - ORNFL**	0.0318	0.0250	0.0406	0.0080
**ORNFL - OPL**	0.1610	0.1479	0.1735	0.0128
**OPL - ETPRS**	0.0618	0.0599	0.0649	0.0027

Q:\Bioptigen\Data\images\site3000254\image\2020.08.24\C200727--017_OS_V_14X14_0_REGAVG0000075.OCT

2/25/2021 10:23:36 AM
